# Serogroup B Meningococcal Disease Vaccine Recommendations at a University, New Jersey, USA, 2016

**DOI:** 10.3201/eid2305.161870

**Published:** 2017-05

**Authors:** Heidi M. Soeters, Jill Dinitz-Sklar, Prathit A. Kulkarni, Jessica R. MacNeil, Lucy A. McNamara, Elizabeth Zaremski, How-yi Chang, Eduardo Lujan, Dan Granoff, Melodee Lasky, Barbara Montana

**Affiliations:** Centers for Disease Control and Prevention, Atlanta, Georgia, USA (H.M. Soeters, P.A. Kulkarni, J.R. MacNeil, L.A. McNamara, H. Chang);; New Jersey Department of Health, Trenton, New Jersey, USA (J. Dinitz-Sklar, P.A. Kulkarni, E. Zaremski, B. Montana);; University of California San Francisco Benioff Children’s Hospital, Oakland, California, USA (E. Lujan, D. Granoff);; Rutgers University, New Brunswick, New Jersey, USA (M. Lasky)

**Keywords:** meningococcal meningitis, serogroup B, universities, meningococcal vaccines, molecular diagnostic techniques, vaccination recommendations, bacteria, bacterial infection, infectious disease, New Jersey, United States, meningitis/encephalitis

## Abstract

In response to a university-based serogroup B meningococcal disease outbreak, the serogroup B meningococcal vaccine Trumenba was recommended for students, a rare instance in which a specific vaccine brand was recommended. This outbreak highlights the challenges of using molecular and immunologic data to inform real-time response.

In 2016, two undergraduate students at a large state university in New Jersey were hospitalized for suspected meningitis. The New Jersey State Public Health Laboratory (Trenton, NJ, USA) identified serogroup B *Neisseria meningitidis* in cerebrospinal fluid specimens from both patients by culture and slide agglutination. Both patients recovered without sequelae.

The isolates were sent to the Centers for Disease Control and Prevention (CDC) Bacterial Meningitis Laboratory for species and serogroup confirmation and whole-genome sequencing. Isolates were confirmed by real-time PCR as serogroup B *N. meningitidis* and were classified by multilocus sequence typing as clonal complex 11, sequence type 11, which is typically associated with serogroups C and W. Whole-genome sequencing revealed that the isolates were genetically indistinguishable from one another but different from all previously characterized meningococcal isolates in the United States.

Two serogroup B meningococcal (MenB) vaccines are licensed in the United States: MenB-4C (Bexsero, GlaxoSmithKline Biologicals, Inc., Philadelphia, PA, USA [*1*]), licensed as a 2-dose series; and MenB-FHbp (Trumenba, Pfizer, Inc., New York, NY, USA [*2*]), licensed as a 2- or 3-dose series. Either Bexsero or the 3-dose series of Trumenba is preferred for outbreak response (*3*). Both MenB vaccines are expected to help protect against most circulating serogroup B meningococcal strains, and in general, no brand preference exists (*3*). However, because the vaccines target different antigens (Table), they are not interchangeable; the same brand must be used for all doses (*5*). The vaccines might also have different effectiveness against specific *N. meningitidis* strains (*5*).

**Table:** Molecular profile and flow cytometry analysis of the university outbreak strain with reference to MenB vaccine antigens, New Jersey, 2016*

Antigen	Outbreak strain	MenB-4C vaccine (Bexsero)	MenB-FHbp vaccine (Trumenba)	Interpretation
FHbp	A22†/2.19‡	B24†/1.1‡	A05†, B01†	The 2 FHbp subfamilies (A and B) are not expected to be cross-reactive. The outbreak strain has a subfamily A FHbp. MenB-FHbp contains FHbp from subfamilies A and B. Although the subfamily A FHbp contained in MenB-FHbp (A05) is not the same peptide allele as that in the outbreak strain (A22), some level of cross-reactivity is expected based on prior testing by the manufacturer (*2*). However, based on flow cytometry, the strain had relatively low expression of FHbp, which would be expected to decrease susceptibility to anti-FHbp bactericidal activity (*4*). MenB-4C contains a subfamily B FHbp, which is not expected to provide protection against the outbreak strain FHbp.
PorA	P1.5–1,10–1	P1.7–2.4	Not included	The PorA present in MenB-4C and in the outbreak strain are 2 different PorA variable region sequence types and are not expected to be cross-reactive; therefore, no protection based on PorA is expected for either vaccine.
NHba	p0020	p0002	Not included	The NHba present in MenB-4C and in the outbreak strain are 2 different peptide alleles. The extent of cross-reactivity is not known. By flow cytometry, low binding to the outbreak strain was observed by using antisera against NHba p0002, but it is unclear whether this low binding is attributable to the difference in sequence between p0020 and p0002, low NHba expression in the outbreak strain, or both. Low binding with NHba p0002 antisera is consistent with decreased susceptibility to anti-NHba bactericidal activity in persons vaccinated with MenB-4C, but the correlation between flow cytometric binding and hSBA response has not been established. No protection based on NHba is expected for MenB-FHbp.
NadA	Negative	2/3.8	Not included	The outbreak strain does not contain NadA; therefore, no protection based on NadA is expected for either vaccine.

*FHbp, factor H binding protein; hSBA, serum bactericidal activity testing using human complement; MenB, serogroup B meningococcal; NadA, Neisserial adhesion A; NHba, Neisserial heparin binding antigen.  †Pfizer classification scheme. ‡GlaxoSmithKline classification scheme.

Whole-genome sequencing identified a mismatch between the antigens in the outbreak strain and those targeted by Bexsero (Table), prompting concern that Bexsero might not provide optimal protection against the outbreak strain. Although the outbreak strain antigens also did not exactly match those included in Trumenba, cross-protection with Trumenba was expected based on prior testing by the manufacturer (Table) (*2*). The outbreak isolates were sent to an independent laboratory for flow cytometry to measure antigen expression and serum bactericidal activity testing using human complement (hSBA) to evaluate whether stored serum from healthy adults previously vaccinated with Bexsero or Trumenba could kill the outbreak strain bacteria (*6*).

By flow cytometry, the outbreak strain had low expression of factor H binding protein and low binding with antisera to the Neisserial heparin binding antigen included in Bexsero (Table) (*7*). Nevertheless, preliminary hSBA results suggested that 2 doses of either Bexsero or Trumenba would provide some short-term protection against the outbreak strain. Among persons with preimmunization titers of <1:4, most had hSBA titers of >1:4 at 1 month after the second dose (13/13 for Bexsero [*7*] and 9/10 for Trumenba). However, by 4 months after the second dose, hSBA titers fell back to <1:4 for some persons vaccinated with Bexsero (4/8) and Trumenba (4/5). The third dose of Trumenba, administered at 6 months after the first dose and 4 months after the second, boosted hSBA titers against outbreak strain bacteria to >1:4 for 9/9 persons 1 month after completion of the 3-dose series. On the basis of consideration of the laboratory data, the best and longest-lasting protection against the outbreak strain was expected with the 3-dose series of Trumenba. Accordingly, the New Jersey Department of Health and the university, with support from CDC, recommended vaccination with 3 doses of Trumenba for ≈35,000 persons at the university.

Serum bactericidal antibodies are used as a serologic correlate of protection for meningococcal vaccines (*8*) and have been correlated with clinical efficacy for serogroup C (*9*). For the purposes of US licensure, the effectiveness of MenB vaccines was inferred by using hSBA. Although hSBA titers probably correlate with protection against serogroup B meningococcal disease, this link has yet to be directly demonstrated through postlicensure effectiveness data.

Although hSBA results informed the vaccination strategy in this outbreak response, this experience underlines the challenges in obtaining hSBA testing and interpreting molecular and immunologic data on meningococcal outbreak strains. The hSBA results for this outbreak strain show that, because of variable protein expression and unknown cross-protection, using the molecular profile of a serogroup B meningococcal strain to reliably predict hSBA response in vaccinated persons is difficult. Additionally, to what extent hSBA results from a limited number of test subjects correspond to real-life protection in a different population is not clear. Furthermore, hSBA testing is time-consuming, a limited amount of serum from immunized humans is available for hSBA testing, and few laboratories are able to routinely perform this testing. These challenges make it difficult to routinely use either molecular typing or hSBA results to guide real-time outbreak response.

This recommendation for a specific brand of MenB vaccine was a rare exception to the general recommendation that either brand of MenB vaccine can help protect persons at increased risk during a serogroup B meningococcal disease outbreak. The use of molecular and immunologic data as potential tools to inform meningococcal outbreak response requires further investigation before being routinely implemented.
